# β-TrCP-mediated ubiquitination and degradation of Dlg5 regulates hepatocellular carcinoma cell proliferation

**DOI:** 10.1186/s12935-019-1029-1

**Published:** 2019-11-15

**Authors:** Dongping Wang, Qi Zhang, Fenfen Li, Chan Wang, Changming Yang, Hong Yu

**Affiliations:** 1 Department of Anesthesiology, The First People’s of Hospital of Jingmen City, Jingmen, Hubei 448000 China; 2 Department of Operation Room, The First People’s of Hospital of Jingmen City, Jingmen, Hubei 448000 China; 3 Department of Nursing, The First People’s of Hospital of Jingmen City, Jingmen, Hubei 448000 China; 4 Department of Neonatology, The First People’s of Hospital of Jingmen City, Jingmen, Hubei 448000 China

**Keywords:** Dlg5, β-TrCP, Wnt/β-catenin signaling, HCC

## Abstract

**Background:**

Discs large homolog 5 (Dlg5) is a member of the membrane-associated guanylate kinase (MAGUK) adaptor family of proteins and its deregulation has been implicated in the malignancy of several cancer types. Dlg5 was down-regulated in hepatocellular carcinoma (HCC) and lower Dlg5 expression was associated with poor survival of HCC patients. However, how to regulate Dlg5 remains largely unknown.

**Methods:**

The co-immunoprecipitation assay was used to determine the interaction between Dlg5 and β-TrCP. The in vivo ubiquitination assay was performed to determine the regulation of Dlg5 by β-TrCP. CCK-8 and colony formation assay were implemented to detect the biological effect of Dlg5 on the growth of HCC cells in vitro. The effect of Dlg5 on HCC tumor growth in vivo was studied in a tumor xenograft model in mice.

**Results:**

Here we report that Dlg5 is regulated by the ubiquitin proteasome system and depletion of either Cullin 1 or β-TrCP led to increased levels of Dlg5. β-TrCP regulated Dlg5 protein stability by targeting it for ubiquitination and subsequent destruction in a phosphorylation-dependent manner. We further demonstrated a crucial role of Ser730 in the non-canonical phosphodegron of Dlg5 in governing β-TrCP-mediated Dlg5 degradation. Importantly, failure to degrade Dlg5 significantly inhibited HCC cells proliferation both in vitro and in vivo.

**Conclusion:**

Collectively, our finding provides a novel molecular mechanism for the negative regulation of Dlg5 by β-TRCP in HCC cells. It further suggests that preventing Dlg5 degradation could be a possible novel strategy for clinical treatment of HCC.

## Background

Liver cancer is the sixth most common cancer in the world and the second malignant tumor of global cancer mortality. It is composed of hepatocellular carcinoma (HCC), intrahepatic cholangiocarcinoma and mixed liver cancer, and about 90% is hepatocellular carcinoma [1]. The pathogenesis of hepatocellular carcinoma (HCC) is extremely complicate, which is the result of multi-factors, multi-genes and multi-stages participation [2, 3]. Thus, the discovery of new molecular drug targets will benefit HCC treatment.

The ubiquitin proteasome pathway (UPP) is critical for protein degradation in eukaryotic organisms. It can selectively degrade many biologically active proteins in cells and participate in the regulation of almost all biological processes, including cell cycle, apoptosis and inflammatory response [4]. Ubiquitination requires the reaction of three enzymes including ubiquitin-activating enzyme E1, ubiquitin-binding enzyme E2, and ubiquitin ligase E3 [5]. E3 ligase plays the most critical role in the entire ubiquitination process by recognizing distinct substrates [6]. The F-box protein family is an important component of the evolutionarily conserved ubiquitin protein ligase complex SCF (Skp1/Cullin1/F-box protein). F-box proteins are able to recruit substrates and promote their ubiquitination and degradation. The F-box protein family has many members, and nearly 70 F-box protein members have been reported in humans [7, 8]. They are classified into three broad categories based on their domain characteristics: the FBXW family with WD40 domain, the FBXL family with leucine-rich domain, and the FBXO family with other domains [9]. However, only a few of these SCF ubiquitin ligases have identified substrates. Noticeably, phosphorylation on specific sites are requested for most SCF substrates which are recognized by most F box proteins [10].

Discs large homolog 5 (Dlg5) is a member of the membrane-associated guanylate kinase (MAGUK) adaptor family of proteins [11]. The down-regulation of Dlg5 has been implicated in the malignancy of HCC [12]. However, how to regulate Dlg5 in HCC is largely unknown. Here we show that the F-box protein β-TrCP regulates Dlg5 protein stability by targeting it for ubiquitination and subsequent destruction in a phosphorylation-dependent manner. The Ser730 phosphorylation of Dlg5 is critical for β-TrCP-mediated Dlg5 degradation, and failure to degrade Dlg5 significantly inhibits HCC cells proliferation both in vitro and in vivo.

## Materials and methods

### Cell culture and reagents

HEK293T cells and hepatocellular carcinoma cell lines SMMC-7721 and HepG2 cells were purchased from Cell Bank of Shanghai Institute of Biological Science (SIBS, CAS, Shanghai, China). Cells were cultured in Dulbecco’s modified Eagle’s medium (DMEM) (Invitrogen), supplemented with 10% FBS (Gibco), 100 units/mL penicillin, and 100 mg/mL streptomycin (Gibco). MLN4924 was purchased from Medkoo. Cycloheximide (CHX) was purchased from Sigma.

### Plasmids

Dlg5, β-TrCP and ubiquitin were amplified from SMMC-7721 cells by polymerase chain reaction and cloned into pcDNA3.1 or pbabe-Flag vector. Five pcDNA3-DN-hCULs-FLAG were gifts from Wade Harper (Addgene plasmid #15818; #15819; #15820; #15821 and # 15822). Dlg5 mutant were generated using QuickChange Site-Directed Mutagenesis Kit (Stratagene). pRK5-HA-Ubiquitin-K48 was a gift from Ted Dawson (Addgene plasmid # 17605; http://n2t.net/addgene:17605; RRID:Addgene_17605). Ub-K48 was then cloned into pcDNA3-his vector. All cDNAs were completely sequenced. All plasmids were transfected into cells using Lipofectamine 2000 (Invitrogen) according to manufacturer instructions.

### RNA interference

All siRNAs were transfected into cells at 100 nM using Lipofectamine RNAimax (Invitrogen). The sequences of β-Trcp siRNA: 5′-AAGUGGAAUUUGUGGAACAUC-3′. The siRNA targeting Cullin1 were purchased from Santa Cruz with cat # sc-35126.

### Construction of stable cell line

Viral supernatants were produced in HEK293T cells co-transfected with the pBabe-Flag-control, pBabe-Flag-Dlg5 WT or pBabe-Flag-Dlg5 S730A plasmids and packaging vectors. Viral supernatants were collected 48 and 72 h after transfection. Filter-sterilized viral supernatants were added to the cells with 10 μg/mL Ploybrane for 48 h and selected with puromycin (2 μg/mL) for a week.

### Western blotting and antibodies

Cells were lysed in SDS loading buffer. The boiled samples were separated by 10% SDS–PAGE and transferred to nitrocellulose (NC) membranes (Whatman, GE Healthcare). The membranes were blocked with 5% milk and incubated with different antibodies overnight at 4, followed by incubation with secondary antibodies. The primary antibodies used in western blotting included anti-Flag M2, anti-HA, anti-Ub, anti-Dlg5 (Sigma-aldrich); anti-β-TrCP (Cell signaling) and anti-Cullin1, anti-SKP1 and anti-β-actin (Santa Cruz).

### Immunoprecipitation (IP)

Cells were lysed in lysis buffer (50 mM Tris–HCl pH 7.5, 150 mM NaCl, 0.5% Nonidet P40, Roche complete EDTA acid-free protease inhibitor cocktail) for 20 min at 4 °C. Lysates were cleared by centrifugation at 12,000*g* for 10 min and the resulting material subjected to IP with each antibody overnight at 4 °C with gentle inversion. Resin containing immune complexes was washed eight times with ice cold lysis buffer and followed by three times Tris-buffered saline (TBS) washes. SDS loading buffer was then added and proteins were eluted with by boiling at 95 °C for 5 min.

### Cell growth and colony formation analysis

SMMC-7721 cells expressing Flag-control, Flag-Dlg5 WT or Flag-Dlg5 S730A. were seeded into six-well plates at 1 × 10^4^/well. Cell numbers were counted by trypan blue staining. For colony formation assays, cells were seeded in a six-well plate at a density of 1000/well and then cultured for 2 weeks. The numbers of colonies containing more than 50 cells were counted by crystal purple staining.

### Xenograft assays

Animal study was approved by Animal Care and Use Committee of the First People’s of Hospital of Jingmen City. Twenty 8-week-old male nude mice were used in this study. All mice were kept in a specific pathogen-free facility. Cells at a density of 5 × 10 ^6^ were suspended in 50 µl of DMEM medium, mixed with Matrigel (Corning; 1:1) and injected into the flanks of male nude mice. Tumor sizes were measured by a caliper. Tumor volumes were calculated using the formula length × width 2 × 1/2. Tumor weights were measured after mice were sacrificed, 3 weeks after injection.

### Statistical analyses

Statistical analysis was performed with GraphPad Prism 7.0 software. Differences between two groups were assessed by Student’s t-test. P values of < 0.05 were considered statistically significant. Statistical significance is displayed as * *P *< 0.05, ** *P* < 0.01, or *** *P *< 0.001.

## Results

### Dlg5 is regulated by the ubiquitin proteasome system via an SCF ubiquitin ligase complex

**Fig. 1:**
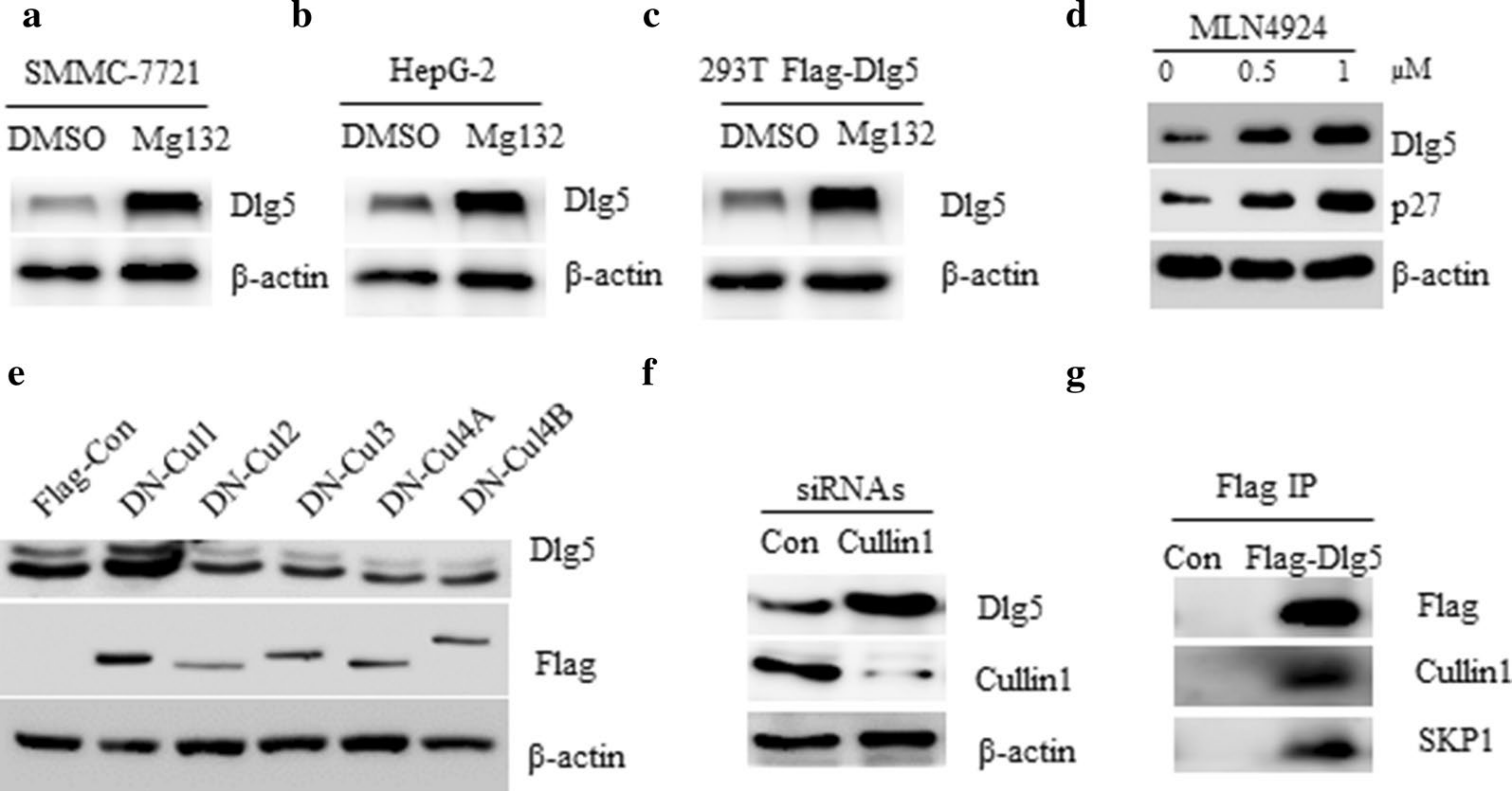
Dlg5 is regulated by the ubiquitin proteasome system via an SCF ubiquitin ligase complex. **a** Western blot analysis of SMMC-7721 cells treated with DMSO or 10 μM MG132 for 4 h. **b** Western blot analysis of HepG-2 cells treated with DMSO or 10 μM MG132 for 4 h. **c** 293T cells were transfected with Flag-Con or Flag-Dlg5 for 36 h, cells were then treated with 10 μM MG132 for 4 h and subjected to western blot analysis with indicated antibodies. **d** Western blot analysis of 293T cells treated with indicated doses of MLN4924 for 4 h. **e** Western blot analysis of 293T cells transfected with DN-Cullin1, DN-Cullin2, DN-Cullin3, DN-Cullin4A or DN-Cullin4B plasmid, respectively. **f** Western blot analysis of SMMC-7721 cells transfected with siRNAs against control or Cullin1. **g** 293T cells were transfected with Flag-Con or Flag-Dlg5 for 36 h, cell lysate was subjected to immunoprecipitation by FlagM2 antibody. Immunoprecipitates were detected by western blot using indicated antibodies

We found that the protein level of Dlg5 was regulated by the ubiquitin proteasome system, as proteasome inhibitor MG132 treatment resulted in the accumulation of endogenous Dlg5 in two HCC cell lines and exogenous expressed Flag-Dlg5 in 293T cells (Fig. 1a–c). As the Cullin-based ubiquitin E3 ligases (CRLs) are usually involved in the degradation of many key proteins during tumorigenesis [13], we then asked whether one of CRLs might be required for the degradation of Dlg5. The activity of Cullins requires NEDD8 conjugation and an investigational NEDD8-activating enzyme inhibitor MLN4924 could prevent NEDD8 modification and inactivate CRLs [14, 15]. To this end, SMMC-7721 cells were treated with MLN4924, and we found that MLN4924 treatment significantly induced the expression of Dlg5 and p27, the latter is a well-known CRL substrate (Fig. 1d). To further test which Cullin is responsible for the degradation of Dlg5, five dominant negative (DN) Cullin members, including Cullin1, Cullin2, Cullin3, Cullin4A and Cullin4B, were expressed into 293T cells, respectively. As shown in Fig. 1e, among five DN-Cullin members, only DN-Cullin1 could significantly induce the expression of Dlg5. Furthermore, silencing the expression of Cullin1 by siRNAs also resulted in the increase of Dlg5 protein (Fig. 1f), suggesting Dlg5 maybe a substrate of the SCF E3 ligase. To test this possibility, 293T cell were transfected with Flag-Dlg5 or Flag-Con for 36 h and Flag-Dlg5 protein complex was immunoprecipitated by Flag M2 beads and subjected to immunoblot detection. We found that both Cullin1 and Skp1 proteins were presented in the precipitated Flag-Dlg5 complex (Fig. 1g). Together, these results indicate that Dlg5 is regulated by the ubiquitin proteasome system by an SCF ubiquitin ligase complex.

### Dlg5 is associated with β-TrCP

**Fig. 2:**
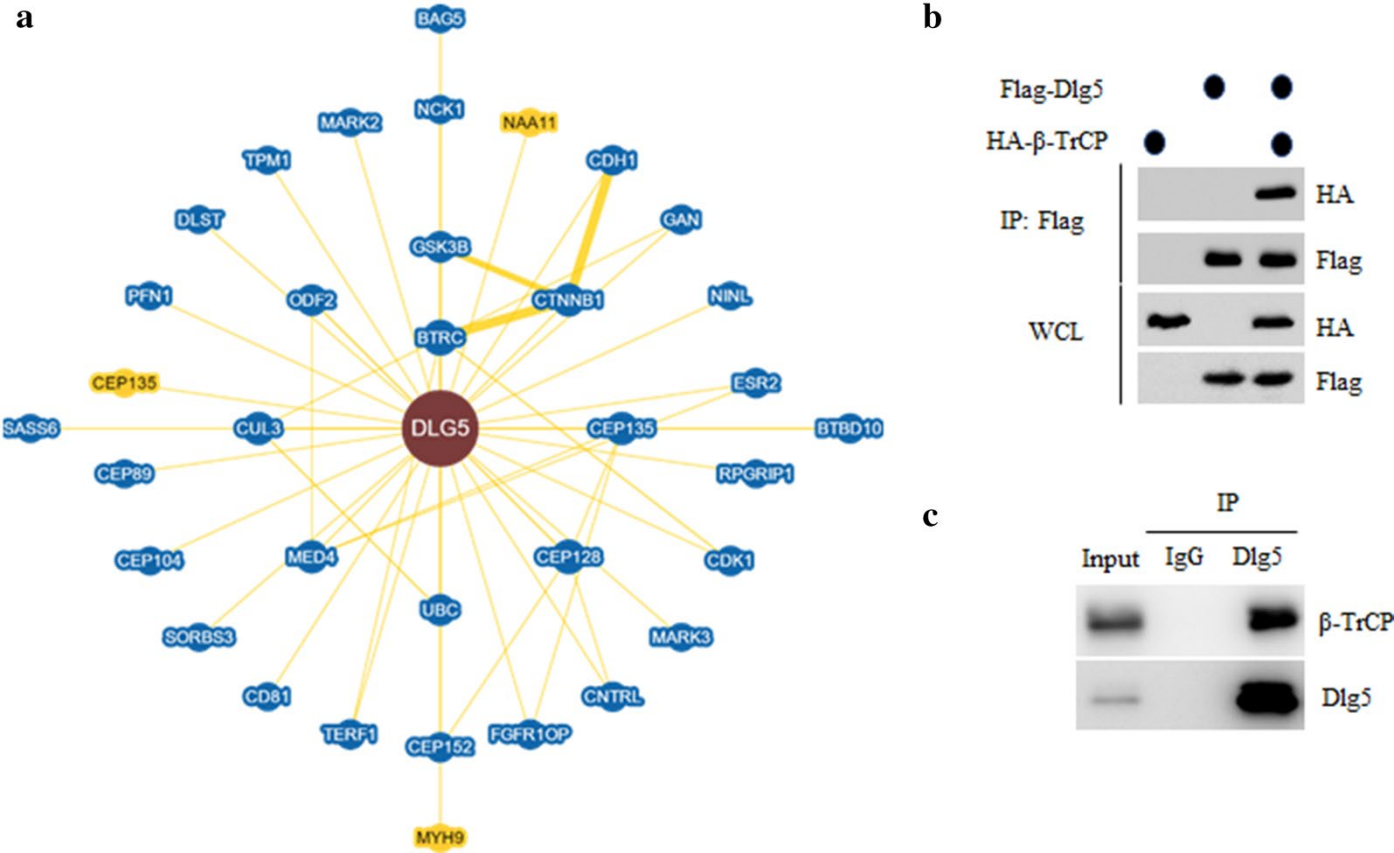
Dlg5 is associated with β-TrCP. **a** The interaction protein network of AEBP2 revealed by the BioGRID database. **b** 293T cell were transfected with Flag-Dlg5 and HA-β-TrCP for 36 h. Flag-Dlg5 protein complex was immunoprecipitated with Flag M2 beads. The Flag-DLG5 immunoprecipitate was detected by western blot using indicated antibodies. **c** SMMC-7721 cell lysate was subjected to immunoprecipitation by anti-Dlg5 antibody. Immunoprecipitates was detected by western blot using indicated antibodies

To better understand how Dlg5 is regulated, we searched the interacting proteins of Dlg5 through the BioGRID database (https://thebiogrid.org/). A total of 35 proteins have been reported to interact with Dlg5 curated by both high throughput and low throughput. Interestingly, we found that the β-TrCP, a substrate recruiting subunit of SCF complex, is listed as interacting protein of Dlg5 by high throughput (Fig. 2a). To confirm the interaction between β-TrCP and Dlg5, 293T cell were transfected with Flag-Dlg5 and HA-β-TrCP for 36 h. Flag-Dlg5 protein complex was immunoprecipitated by Flag M2 beads and subjected to immunoblot with anti-Flag or anti-HA antibodies, respectively. As expected, HA-β-TrCP was readily detected in Flag-Dlg5 immunoprecipitate (Fig. 2b). Moreover, the interaction between endogenous β-TrCP and Dlg5 was further confirmed in SMMC-7721 cells (Fig. 2c). Together, these data indicate that Dlg5 is associated with β-TrCP protein.

### β-TrCP regulates the stability and ubiquitination of Dlg5

**Fig. 3:**
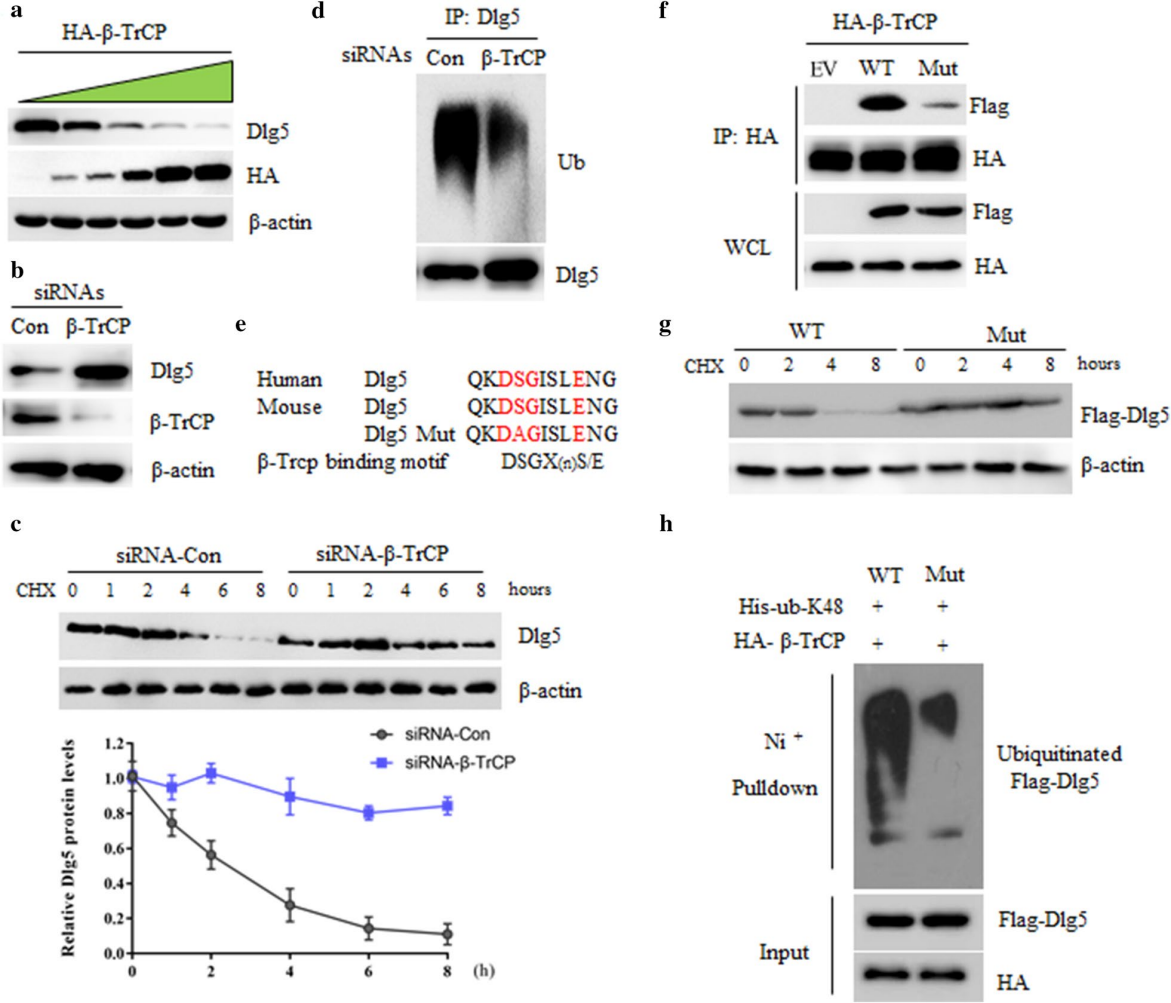
β-TrCP regulates the stability and ubiquitination of Dlg5. **a** Western blot analysis of SMMC-7721 cells transfected with indicated doses of Flag-β-TrCP. **b** Western blot analysis of SMMC-7721 cells transfected with siRNAs against control or β-TrCP. **c** SMMC-7721 cells transfected with siRNAs against control or β-TrCP for 24 h, then 20 μg/ml cycloheximide (CHX) was added for the indicated time course. Cell extracts were subjected to western blot with the indicated antibodies. **d** SMMC-7721 cells transfected with siRNAs against control or β-TrCP for 36 h, cell lysate was subjected to immunoprecipitation by anti-Dlg5 antibody. Immunoprecipitates was detected by western blot using indicated antibodies. **e** Alignment of amino acids corresponding to the DSGxxxE sequence with Dlg5 orthologs. **f** 293T cells were co-transfected Flag-β-TrCP with HA-Dlg5 WT or HA-Dlg5 S730A for 36 h, cell lysate was subjected to immunoprecipitation by anti-Flag antibody. Immunoprecipitates was detected by western blot using indicated antibodies. **g** 293T cells transfected with indicated plasmids for 36 h, cell lysate was subjected to western blot detection. **h** 293T cells were co-transfected Flag-β-TrCP and his-K48-ub with HA-Dlg5 WT or HA-Dlg5 S730A for 36 h, cell lysate was subjected to immunoprecipitation by Ni+ purification. Immunoprecipitate was detected by western blot using indicated antibodies

To investigate whether β-TrCP can regulate Dlg5 protein, we overexpressed β-TrCP into SMMC-7721 cells and found that overexpression of β-TrCP resulted in a marked reduction of Dlg5 levels in a dose dependent manner (Fig. 3a). Next, we used siRNA to interfere with the expression of β-TrCP in SMMC-7721 cells, and found that silencing of β-TrCP resulted in the increase of Dlg5 protein (Fig. 3b). This increase is due to the reduced degradation of Dlg5 protein, as shown by the increase in Dlg5 half-life (Fig. 3c). Consistent with these data, silencing of β-TrCP decreased the ubiquitination form of Dlg5 (Fig. 3d). Usually, β-TrCP targets substrates for ubiquitination by recognizing a phosphodegron on them [16]. We found that Dlg5 contains a conserved β-TrCP degron (DSGxxxE) (Fig. 3e). Next, we asked whether this putative degron is sufficient for β-TrCP binding. To this end, we generated a mutant in which serine 730 was mutated to alanine (S730A), and test its binding activity to β-TrCP. In contrast to Wild-type (WT) Dlg5, the interaction between Dlg5 S730A mutant and β-TrCP was greatly impaired, if not completely (Fig. 3f), suggesting the phosphorylation of serine residues within DSGxxxE is critical for β-TrCP recognition. In concert with this, the half-life of Dlg5 S730A mutant was increased when compared with Dlg5 WT (Fig. 3g) and the K48 ubiquitination form of Dlg5 S730A mutant was decreased (Fig. 3h). Together, these data suggested that β-TrCP regulated the stability and ubiquitination of Dlg5 in a phosphorylation-dependent manner.

### Failure to degrade Dlg5 significantly inhibited HCC cells proliferation

**Fig. 4:**
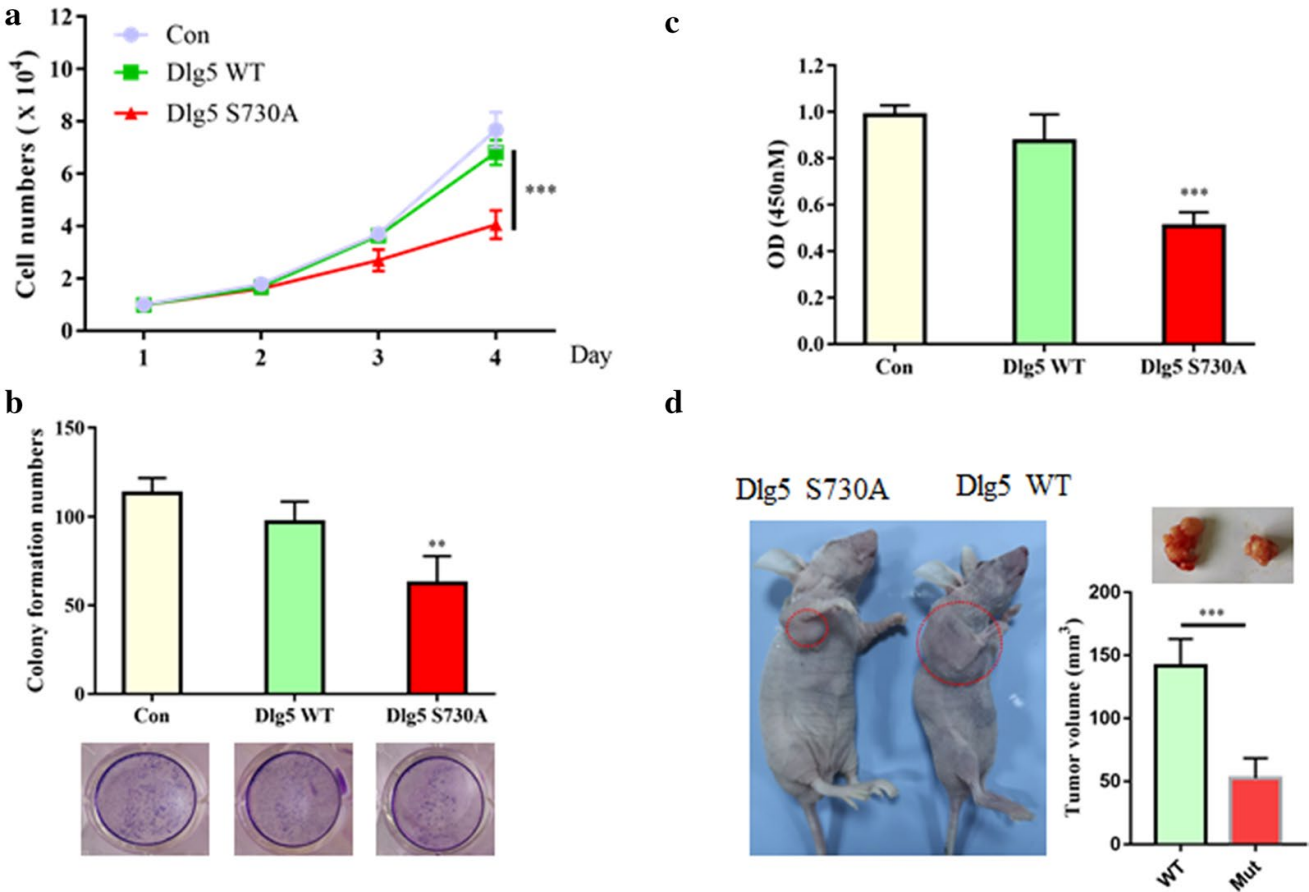
Failure to degrade Dlg5 significantly inhibited HCC cells proliferation. **a** The cell growth curves of control, Dlg5 WT or Dlg5 S730A cells for 4 days. **b** The cell viability of control, Dlg5 WT or Dlg5 S730A cells were measured by CCK-8 assay. Data represents mean ± SD. Results were averaged from three independent experiments. **c** Clone formation of control, Dlg5 WT or Dlg5 S730A cells were determined by soft agar colony formation assay. 1000 cells were seeded into each well, and cultured for another 2 weeks. Cells were stained with crystal violet and then counted. **d** Each nude mouse was subcutaneously injected with 5 × 10^6^ cells stably expressing Dlg5 WT or Dlg5 S730A for 3 weeks. n = 10 for nude mice injected with Dlg5 WT or Dlg5 S730A cells, separately

To investigate whether failure to degrade Dlg5 affects HCC cells proliferation, we analyzed SMMC-7721 cells expressing Flag-tagged Dlg5 WT or Flag-tagged Dlg5 S730A. As expected, overexpression of Dlg5 WT did not affect SMMC-7721 cells proliferation, however cells expressing Flag-tagged Dlg5 S730A showed decreased proliferation activity by both cell number counting and CCK-8 assays (Fig. 4a, b). Moreover, cells expressing Flag-tagged Dlg5 S730A exhibited significant deceased colony formation ability when compared with either control cells or cells expressing Dlg5 WT (Fig. 4c). We also used nude mice model to investigate whether failure to degrade Dlg5 affected cells proliferation in vivo. Four weeks old BALB/c nude mice were subcutaneously injected with 5 × 10^6^ SMMC-7721 cells either expressing Flag-tagged Dlg5 WT or Flag-tagged Dlg5 S730A. As expected, SMMC-7721 cells stable expressing of a non-degradable Dlg5 S730A mutant dramatically decreased tumorigenesis, as the tumor volume was significantly decreased compared with Dlg5 WT group (Fig. 4d). Together, these data suggested that failure to degrade Dlg5 significantly inhibited HCC cells proliferation both in vitro and in vivo.

## Discussion

Dlg5 is an important protein that maintains the polarity of epithelial cells and is essential for the maintenance of adhesion junctions and the formation of epithelial polarity [17]. Dlg5 knockout mice exhibited cerebral aqueduct occlusion, severe brain edema, renal cysts, emphysema-like lesions, and half of these mice were dead during perinatal [18]. The main cause of these lesions is the loss of cell adhesion junctions and epithelial polarity. Cadherin family is critical for cell adhesion junctions and epithelial polarity maintenance [19]. Dlg5 is involved in the correct localization of N-cadherin to the cell membrane [20]. Deletion of Dlg5 will result in the failure of N-cadherin localization, which in turn destroys the adhesion junction between cells, ultimately leading to the failure of epithelial cell polarity maintenance [20].

The loss of polar proteins leads to the failure of epithelial cell polarity maintenance and is closely related to tumorigenesis [21]. The down-regulation of Dlg5 has been implicated in the malignancy of breast, prostate, bladder cancers and HCC [12, 22–24]. It has been reported that lower Dlg5 expression is correlated with advanced stages of HCC [12]. Thus, to understand how Dlg5 is regulated might shed some light on the development of HCC. It has been shown that the methylation of the promoter region of DLG5 may be associated with its low expression in some tumor tissues [23]. Here, our data reveal a previous unknown molecular mechanism for the posttranscriptional regulation of Dlg5 protein in HCC cells.

Firstly, we have discovered that Dlg5 undergoes sustained ubiquitination degradation in cells, and this process requires a functional SCF E3 ligase. By searching a large protein–protein interaction database, we found that Dlg5 is a β-TrCP interacting protein. Through several biochemical analysis, we verified the binding of β-TrCP to Dlg5 and found that β-TrCP promoted the proteasome-dependent degradation of Dlg5. Importantly, we found that the phosphorylation of S730 is required for Dlg5 ubiquitination and degradation. Dlg5 S730A mutant, which cannot be recognized by β-TrCP, the protein stability of which was significantly enhanced, and the ubiquitination was reduced. It has been reported that overexpression of Dlg5 does not significantly affect the growth of HCC SK-Hep1 cells [12], and we recaptured this phenomenon in SMMC-7721 cells overexpressing Dlg5 WT. However, we found that overexpression of Dlg5 S730A significantly inhibited the growth of SMMC-7721 cells. The discrepancy could be explained by the different protein stability between Dlg5 WT and Dlg5 S730A. We believe that Dlg5 WT is still regulated by β-TrCP-mediated ubiquitination and degradation, while Dlg5 S730A is not, and therefore enhanced its ability to inhibit cell proliferation. Consistent with these in vitro data, we also found that cells stably expressing Dlg5 S730A significantly inhibited tumor growth in nude mice than Dlg5 WT. Given the critical role of Dlg5 to maintain cell polarity, cells stably expressing Dlg5 S730A mutations will continue to maintain cell polarity and inhibit tumor development.

## Conclusion

Our study is the first to show that Dlg5 is precisely regulated by different posttranscriptional ways and phosphorylation of Dlg5 at S730A is required for its ubiquitination and degradation. Our finding further provides a novel molecular mechanism for the negative regulation of Dlg5 by β-TRCP in HCC cells. Interference with this regulatory effect on Dlg5 significantly inhibited HCC cells proliferation. Thus, our data suggest that preventing Dlg5 degradation could be a possible novel strategy for clinical treatment of HCC.

## Data Availability

Please contact corresponding author for data requests.

## Abbreviations

CRLs: Cullin-based ubiquitin E3 ligases
Dlg5: discs large homolog 5
HCC: hepatocellular carcinoma
IP: immunoprecipitation
MAGUK: membrane-associated guanylate kinase
SCF: Skp1/Cullin1/F-box protein
UPP: ubiquitin proteasome pathway
